# Gender disparities among authors of retracted publications in medical journals: A cross-sectional study

**DOI:** 10.1371/journal.pone.0335059

**Published:** 2025-11-19

**Authors:** Paul Sebo

**Affiliations:** University Institute for Primary Care (IuMFE), University of Geneva, Geneva, Switzerland; Rajagiri College of Social Sciences, INDIA

## Abstract

**Background:**

Gender disparities in scientific authorship are well documented, yet little is known about gender representation among authors of retracted publications.

**Methods:**

We analyzed 878 retracted publications from 131 high-impact medical journals across nine clinical disciplines (anesthesiology, dermatology, general internal medicine, gynecology/obstetrics, neurology, oncology, pediatrics, psychiatry, and radiology). Gender was inferred using Gender API for all, first, and last authors. Two analytic samples were constructed based on prediction confidence thresholds (≥60% and ≥70%). We examined gender distribution across authorship positions, number of retractions per author, and disciplinary representation. Wilcoxon rank-sum and chi-squared tests were used to assess group differences. Gender proportions were compared with publication benchmarks from 2008–2017, restricting retraction data to the same period for comparability.

**Results:**

Among 4,136 authors, 3,909 had full first names, and gender could be assigned to 3,865 (98.9%). In the sample with prediction confidence ≥60% (n = 3,743), 863 (23.1%) were identified as women. They accounted for 16.5% (123/747) of first and 12.7% (87/687) of last authors. They had significantly fewer retractions per author and were less likely to have >5 retractions (all authors: 3 women [8.1%] vs 34 men [91.9%], p < 0.001). Across most disciplines, their representation was below publication benchmarks. Dermatology (retractions = 80.0%, publications = 48.9–51.8%) and radiology (retractions = 40.0%, publications = 31.0-36.8%) were exceptions among first authors, while pediatrics (retractions = 50.0%, publications = 37.0%−42.6%) was an exception among last authors, though all based on small numbers.

**Conclusions:**

Women are markedly underrepresented among authors of retracted publications, particularly in cases involving multiple retractions. Further research is needed to clarify underlying mechanisms.

## Introduction

The retraction of scientific articles serves a crucial function in correcting the academic record and is widely regarded as a key mechanism for preserving the integrity of biomedical literature [1–4]. Retractions may result from a range of issues, including honest error, publisher mistakes, or research misconduct such as fabrication, falsification, and plagiarism [2–9]. Although retractions remain relatively rare [2,3,8,9], their impact can be substantial, particularly in high-impact medical journals where retracted findings may have already influenced clinical practice or policy.

A growing body of research has examined the characteristics of retracted publications, including country of origin [2–4,6,10–12], discipline [3,4,6,11], and reason for retraction [2–9]. Fewer studies have explored the demographics of authors, particularly gender [7,13–16], and those that did reveal important limitations in scope, methodology, and data completeness. To our knowledge, only three studies benchmarked gender distribution in retractions against overall publication output [7,15,16].

In a preliminary analysis of 438 retracted publications in medical journals, we found a marked underrepresentation of women among first and last authors, especially in misconduct-related cases [7]. This analysis was limited by a relatively small retraction sample and a mismatch in time frames, with retractions covering 2003–2022 and publication data only 2008–2017.

Two recent studies have extended this line of inquiry beyond medicine. Zheng et al. combined Web of Science (WoS) retraction and publication data with retraction reasons from Retraction Watch Database (RWD) and found that male authors generally had higher retraction rates than female authors, particularly for misconduct [15]. However, patterns varied by field: male authors experienced significantly higher retraction rates in biomedical and health sciences, as well as in life and earth sciences, whereas female authors had higher retraction rates in mathematics and computer science. The main analyses focused on first authors, but similar patterns were observed for corresponding authors. Yet, the study’s validity is limited by: (1) reliance on WoS, which is less comprehensive than RWD [1]; (2) restriction to first authors for the main analyses; (3) substantial missingness in gender attribution (only 53% of retracted articles and 77% of non-retracted articles), raising concerns about selection bias and representativeness; and (4) possible bias from a non-comparable reference group, as gender could be determined for a much higher proportion of non-retracted authors than for retracted authors. The low gender match rates reported by Zheng et al. are likely due to their use of a stringent ≥90% confidence threshold for gender inference—based on a combination of tools (Gender API, Genderize.io, and Gender Guesser)—which naturally leads to more names being labeled ‘unknown’ and excluded from analysis [17].

Maddi et al. combined OpenAlex publication data with retraction data from RWD and found that retraction risk varied by team composition: mixed-gender teams were more likely to face retractions than all-male or all-female teams, whereas individually authored publications were less likely to be retracted [16]. Larger teams had a lower likelihood of retraction, while medium-sized teams (3–10 authors) faced a higher risk. Retraction reasons also differed by gender, with male-led publications more often retracted for serious ethical violations and female-led publications more often retracted for procedural errors or updates in rapidly evolving fields. The study’s limitations include: (1) reliance on two data sources with different coverage, indexing practices, and metadata completeness, introducing potential systematic bias; (2) gender attribution being possible for only 67% of authors in the publication dataset (not reported for the retraction dataset), which may skew results; and (3) use of genderize.io without specifying the probability threshold, reducing reproducibility and interpretability.

Taken together, these studies suggest that women are underrepresented among authors of retracted publications, particularly in misconduct-related cases, mirroring broader patterns of gender disparity in research. The underrepresentation of women in science is well documented [18–32]. They remain a minority in senior academic roles [21,23] and hold fewer authorship [18,19,23,32] and editorial leadership positions in scientific journals [31]. This imbalance may influence not only publishing opportunities but also exposure to scrutiny and patterns of retraction.

Differences in the indexing of retracted publications across major bibliographic databases complicate efforts to analyze retraction patterns [1,33–36]. In a recent study, we demonstrated that while RWD outperformed PubMed and the WoS Core Collection in identifying retracted publications, none of the databases offered complete coverage [1]. The present study builds upon this prior work by using the same dataset of 878 retracted publications from 131 high-impact medical journals across nine clinical disciplines to examine gender disparities among authors of retracted articles.

We aim to quantify the gender distribution of all authors, first authors, and last authors of retracted publications, and to compare these distributions with established benchmarks for female authorship in biomedical literature [18]. We also assess whether gender differences are more pronounced in misconduct-related retractions and examine variation across medical specialties. By highlighting these patterns, our study contributes to the broader conversation on equity, transparency, and trust in scientific publishing.

Based on previous findings, we hypothesize that women are underrepresented among the authors of retracted publications. We further expect that this underrepresentation is more pronounced in misconduct-related retractions, and that gender disparities vary across medical disciplines. Finally, we hypothesize that the proportion of retracted publications with female first or last authors is lower than the baseline proportion of female authors observed in biomedical publishing at large.

## Methods

### Study design and objective

This cross-sectional study aimed to evaluate gender disparities among authors of retracted publications in high-impact medical journals. Specifically, we assessed the gender distribution of authors whose articles were retracted, both overall and in cases involving research misconduct. We analyzed gender representation among all authors, first authors, and last authors of retracted publications. We also assessed variation in gender representation across disciplines and compared the gender composition of retracted articles to known publication patterns in biomedical research.

This study builds on a previously developed dataset designed to compare the performance of RWD, PubMed, and the WoS Core Collection in identifying retracted publications in medicine [1]. The dataset included all retracted publications indexed up to December 15, 2024, in 131 high-impact medical journals across nine clinical disciplines: anesthesiology, dermatology, general internal medicine, neurology, obstetrics/gynecology, oncology, pediatrics, psychiatry, and radiology/nuclear medicine/medical imaging (Table 1). Journals were selected from Clarivate’s 2023 Journal Citation Reports (JCR) as the 15 with the highest impact factor per discipline. Overlaps were resolved by allowing journals to appear in two disciplines when appropriate, resulting in 131 unique journals.

**Table 1:** Journals included in the study, grouped by discipline and ranked by 2023 Journal Citation Reports (JCR) impact factor.

Abbreviated journal name (PubMed)	ISSN	e-ISSN	Discipline	2023 JCR Impact Factor
*Anesthesiology*	0003-3022	1528-1175	ANESTHESIOLOGY	9.3
*Br J Anaesth*	0007-0912	1471-6771	ANESTHESIOLOGY	9.1
*Anaesthesia*	0003-2409	1365-2044	ANESTHESIOLOGY	7.5
*Pain*	0304-3959	1872-6623	ANESTHESIOLOGY	5.9
*Reg Anesth Pain Med*	1098-7339	1532-8651	ANESTHESIOLOGY	5.1
*J Clin Anesth*	0952-8180	1873-4529	ANESTHESIOLOGY	5.0
*Best Pract Res Clin Anaesthesiol*	1521-6896	1878-1608	ANESTHESIOLOGY	4.7
*Anesth Analg*	0003-2999	0003-2999	ANESTHESIOLOGY	4.6
*Eur J Anaesthesiol*	0265-0215	1365-2346	ANESTHESIOLOGY	4.2
*Korean J Anesthesiol*	2005-6419	2005-7563	ANESTHESIOLOGY	4.2
*Anaesth Crit Care Pain Med*	2352-5568	2352-5568	ANESTHESIOLOGY	3.7
*Eur J Pain*	1090-3801	1532-2149	ANESTHESIOLOGY	3.5
*Can J Anaesth*	0832-610X	1496-8975	ANESTHESIOLOGY	3.4
*Pain Med*	1526-2375	1526-4637	ANESTHESIOLOGY	2.9
*Indian J Anaesth*	0019-5049	0976-2817	ANESTHESIOLOGY	2.9
*J Am Acad Dermatol*	0190-9622	1097-6787	DERMATOLOGY	12.8
*JAMA Dermatol*	2168-6068	2168-6084	DERMATOLOGY	11.5
*Br J Dermatol*	0007-0963	1365-2133	DERMATOLOGY	11.0
*Am J Clin Dermatol*	1175-0561	1179-1888	DERMATOLOGY	8.6
*J Eur Acad Dermatol Venereol*	0926-9959	1468-3083	DERMATOLOGY	8.5
*Burns Trauma*	2321-3868	2321-3876	DERMATOLOGY	6.3
*J Invest Dermatol*	0022-202X	1523-1747	DERMATOLOGY	5.9
*Adv Wound Care*	2162-1918	2162-1934	DERMATOLOGY	5.8
*J Dtsch Dermatol Ges*	1610-0379	1610-0387	DERMATOLOGY	5.6
*Psoriasis Targets Ther*	N/A	2230-326X	DERMATOLOGY	5.2
*Contact Dermatitis*	0105-1873	1600-0536	DERMATOLOGY	4.8
*Mycoses*	0933-7407	1439-0507	DERMATOLOGY	4.1
*Dermatitis*	1710-3568	2162-5220	DERMATOLOGY	4.0
*Pigment Cell Melanoma Res*	1755-1471	1755-148X	DERMATOLOGY	3.9
*J Dermatol Sci*	0923-1811	1873-569X	DERMATOLOGY	3.8
*Lancet*	0140-6736	1474-547X	MEDICINE, GENERAL & INTERNAL	98.4
*N Engl J Med*	0028-4793	1533-4406	MEDICINE, GENERAL & INTERNAL	96.3
*BMJ*	0959-535X	1756-1833	MEDICINE, GENERAL & INTERNAL	93.7
*Nat Rev Dis Primers*	2056-676X	2056-676X	MEDICINE, GENERAL & INTERNAL	79.0
*JAMA*	0098-7484	1538-3598	MEDICINE, GENERAL & INTERNAL	63.5
*Lancet Digit Health*	N/A	2589-7500	MEDICINE, GENERAL & INTERNAL	23.8
*JAMA Intern Med*	2168-6106	2168-6114	MEDICINE, GENERAL & INTERNAL	22.3
*Ann Intern Med*	0003-4819	1539-3704	MEDICINE, GENERAL & INTERNAL	19.6
*Mil Med Res*	2095-7467	2054-9369	MEDICINE, GENERAL & INTERNAL	16.7
*J R Soc Med*	0141-0768	1758-1095	MEDICINE, GENERAL & INTERNAL	16.3
*CMAJ*	0820-3946	1488-2329	MEDICINE, GENERAL & INTERNAL	12.9
*JAMA Netw Open*	2574-3805	2574-3805	MEDICINE, GENERAL & INTERNAL	10.5
*PLoS Med*	1549-1277	1549-1676	MEDICINE, GENERAL & INTERNAL	10.5
*BMJ Evid Based Med*	2515-446X	2515-4478	MEDICINE, GENERAL & INTERNAL	9.8
*EClinicalMedicine*	N/A	2589-5370	MEDICINE, GENERAL & INTERNAL	9.6
*Lancet Neurol*	1474-4422	1474-4465	CLINICAL NEUROLOGY	46.6
*Nat Rev Neurol*	1759-4758	1759-4766	CLINICAL NEUROLOGY	28.2
*JAMA Neurol*	2168-6149	2168-6157	CLINICAL NEUROLOGY	20.9
*Neuro Oncol*	1522-8517	1523-5866	CLINICAL NEUROLOGY	16.4
*Alzheimers Dement*	1552-5260	1552-5279	CLINICAL NEUROLOGY	13.1
*Brain*	0006-8950	1460-2156	CLINICAL NEUROLOGY	11.9
*Sleep Med Rev*	1087-0792	1532-2955	CLINICAL NEUROLOGY	11.2
*Acta Neuropathol*	0001-6322	1432-0533	CLINICAL NEUROLOGY	9.3
*J Neurol Neurosurg Psychiatry*	0022-3050	1468-330X	CLINICAL NEUROLOGY	8.8
*JPAD*	2274-5807	2426-0266	CLINICAL NEUROLOGY	8.5
*Neurology*	0028-3878	1526-632X	CLINICAL NEUROLOGY	8.4
*Neurol Neuroimmunol Neuroinflamm*	2332-7812	2332-7812	CLINICAL NEUROLOGY	8.3
*Ann Neurol*	0364-5134	1531-8249	CLINICAL NEUROLOGY	8.1
*Alzheimers Res Ther*	N/A	1758-9193	CLINICAL NEUROLOGY	8.0
*Stroke*	0039-2499	1524-4628	CLINICAL NEUROLOGY	7.9
*Hum Reprod Update*	1355-4786	1460-2369	OBSTETRICS & GYNECOLOGY	14.8
*Am J Obstet Gynecol*	0002-9378	1097-6868	OBSTETRICS & GYNECOLOGY	8.7
*Hum Reprod Open*	N/A	2399-3529	OBSTETRICS & GYNECOLOGY	8.3
*Fertil Steril*	0015-0282	1556-5653	OBSTETRICS & GYNECOLOGY	6.6
*Ultrasound Obstet Gynecol*	0960-7692	1469-0705	OBSTETRICS & GYNECOLOGY	6.1
*Hum Reprod*	0268-1161	1460-2350	OBSTETRICS & GYNECOLOGY	6.0
*Obstet Gynecol*	0029-7844	0029-7844	OBSTETRICS & GYNECOLOGY	5.8
*Breast*	0960-9776	1532-3080	OBSTETRICS & GYNECOLOGY	5.7
*Obstet Gynecol Surv*	0029-7828	1533-9866	OBSTETRICS & GYNECOLOGY	5.2
*BJOG*	1470-0328	1471-0528	OBSTETRICS & GYNECOLOGY	4.8
*Gynecol Oncol*	0090-8258	1095-6859	OBSTETRICS & GYNECOLOGY	4.5
*Update Int J Gynecol Cancer*	1048-891X	1525-1438	OBSTETRICS & GYNECOLOGY	4.5
*Women Birth*	1871-5192	1878-1799	OBSTETRICS & GYNECOLOGY	4.4
*Breast Cancer*	1340-6868	1880-4233	OBSTETRICS & GYNECOLOGY	4.0
*Best Pract Res Clin Obstet Gynaecol*	1521-6934	1532-1932	OBSTETRICS & GYNECOLOGY	3.9
*CA Cancer J Clin*	0007-9235	1542-4863	ONCOLOGY	521.6
*Nat Rev Clin Oncol*	1759-4774	1759-4782	ONCOLOGY	81.1
*Nat Rev Cancer*	1474-175X	1474-1768	ONCOLOGY	72.5
*Ann Oncol*	0923-7534	1569-8041	ONCOLOGY	56.7
*Cancer Cell*	1535-6108	1878-3686	ONCOLOGY	48.8
*J Clin Oncol*	0732-183X	1527-7755	ONCOLOGY	42.1
*Lancet Oncol*	1470-2045	1474-5488	ONCOLOGY	41.6
*Cancer Discov*	2159-8274	2159-8290	ONCOLOGY	30.6
*J Hematol Oncol*	N/A	1756-8722	ONCOLOGY	29.9
*Mol Cancer*	N/A	1476-4598	ONCOLOGY	27.7
*Nat Cancer*	N/A	2662-1347	ONCOLOGY	23.5
*JAMA Oncol*	2374-2437	2374-2445	ONCOLOGY	22.3
*J Thorac Oncol*	1556-0864	1556-1380	ONCOLOGY	21.1
*Cancer Commun*	N/A	2523-3548	ONCOLOGY	20.1
*Neuro Oncol*	1522-8517	1523-5866	ONCOLOGY	16.4
*JAMA Pediatr*	2168-6203	2168-6211	PEDIATRICS	24.7
*Lancet Child Adolesc Health*	2352-4642	2352-4642	PEDIATRICS	19.9
*J Am Acad Child Adolesc Psychiatry*	0890-8567	1527-5418	PEDIATRICS	9.2
*Child Adolesc Ment Health*	1475-357X	1475-3588	PEDIATRICS	6.8
*Pediatrics*	0031-4005	1098-4275	PEDIATRICS	6.2
*Eur Child Adolesc Psychiatry*	1018-8827	1435-165X	PEDIATRICS	6.0
*J Adolesc Health*	1054-139X	1879-1972	PEDIATRICS	5.5
*Paediatr Respir Rev*	1526-0542	1526-0550	PEDIATRICS	4.7
*Arch Dis Child*	0003-9888	1468-2044	PEDIATRICS	4.4
*Pediatr Allergy Immunol*	0905-6157	1399-3038	PEDIATRICS	4.3
*Pediatr Crit Care Med*	1529-7535	1947-3893	PEDIATRICS	4.1
*Int J Neonatal Screen*	N/A	2409-515X	PEDIATRICS	4.0
*Arch Dis Child Fetal Neonatal Ed*	1359-2998	1468-2052	PEDIATRICS	3.9
*J Pediatr*	0022-3476	1097-6833	PEDIATRICS	3.9
*Pediatr Diabetes*	1399-543X	1399-5448	PEDIATRICS	3.9
*World Psychiatry*	1723-8617	2051-5545	PSYCHIATRY	60.5
*Lancet Psychiatry*	2215-0374	N/A	PSYCHIATRY	30.8
*JAMA Psychiatry*	2168-622X	2168-6238	PSYCHIATRY	22.5
*Psychother Psychosom*	0033-3190	1423-0348	PSYCHIATRY	16.3
*Am J Psychiatry*	0002-953X	1535-7228	PSYCHIATRY	15.1
*Mol Psychiatry*	1359-4184	1476-5578	PSYCHIATRY	9.6
*Biol Psychiatry*	0006-3223	1873-2402	PSYCHIATRY	9.6
*J Am Acad Child Adolesc Psychiatry*	0890-8567	1527-5418	PSYCHIATRY	9.2
*Ment Illn*	2036-7457	2036-7465	PSYCHIATRY	9.0
*J Neurol Neurosurg Psychiatry*	0022-3050	1468-330X	PSYCHIATRY	8.8
*Brain Behav Immun*	0889-1591	1090-2139	PSYCHIATRY	8.8
*Br J Psychiatry*	0007-1250	1472-1465	PSYCHIATRY	8.8
*Curr Opin Psychiatry*	0951-7367	1473-6578	PSYCHIATRY	7.5
*CNS Drugs*	1172-7047	1179-1934	PSYCHIATRY	7.4
*Eur Psychiatry*	0924-9338	1778-3585	PSYCHIATRY	7.2
*JACC Cardiovasc Imaging*	1936-878X	1876-7591	RADIOLOGY, NUCLEAR MEDICINE & MEDICAL IMAGING	12.8
*Radiology*	0033-8419	N/A	RADIOLOGY, NUCLEAR MEDICINE & MEDICAL IMAGING	12.1
*Med Image Anal*	1361-8415	1361-8423	RADIOLOGY, NUCLEAR MEDICINE & MEDICAL IMAGING	10.7
*Clin Nucl Med*	0363-9762	1536-0229	RADIOLOGY, NUCLEAR MEDICINE & MEDICAL IMAGING	10.0
*Radiol Med*	0033-8362	1826-6983	RADIOLOGY, NUCLEAR MEDICINE & MEDICAL IMAGING	9.7
*J Nucl Med*	0161-5505	1535-5667	RADIOLOGY, NUCLEAR MEDICINE & MEDICAL IMAGING	9.1
*IEEE Trans Med Imaging*	0278-0062	1558-254X	RADIOLOGY, NUCLEAR MEDICINE & MEDICAL IMAGING	8.9
*Eur J Nucl Med Mol Imaging*	1619-7070	1619-7089	RADIOLOGY, NUCLEAR MEDICINE & MEDICAL IMAGING	8.6
*Radiol Artif Intell*	2638-6100	2638-6100	RADIOLOGY, NUCLEAR MEDICINE & MEDICAL IMAGING	8.1
*Photoacoustics*	2213-5979	2213-5979	RADIOLOGY, NUCLEAR MEDICINE & MEDICAL IMAGING	7.1
*Invest Radiol*	0020-9996	1536-0210	RADIOLOGY, NUCLEAR MEDICINE & MEDICAL IMAGING	7.0
*Eur Heart J Cardiovasc Imaging*	2047-2404	2047-2412	RADIOLOGY, NUCLEAR MEDICINE & MEDICAL IMAGING	6.7
*Circ Cardiovasc Imaging*	1941-9651	1942-0080	RADIOLOGY, NUCLEAR MEDICINE & MEDICAL IMAGING	6.5
*Int J Radiat Oncol Biol Phys*	0360-3016	1879-355X	RADIOLOGY, NUCLEAR MEDICINE & MEDICAL IMAGING	6.4
*Ultrasound Obstet Gynecol*	0960-7692	1469-0705	RADIOLOGY, NUCLEAR MEDICINE & MEDICAL IMAGING	6.1

### Retraction data sources and extraction

Retractions were identified by searching three databases: RWD, PubMed, and the WoS Core Collection. Searches were conducted using journal names, ISSNs, and eISSNs. Retractions were included if indexed in any of the three databases. Retrieved records were cleaned and de-duplicated using PubMed IDs (PMIDs) and article titles. When PMIDs were unavailable, matching was done manually using article metadata. The final dataset represents the union of retractions retrieved from the three databases.

We used metadata from RWD to classify retractions as related or unrelated to research misconduct. Articles were classified as misconduct-related if the reason for retraction included fabrication or falsification of data, images, or results; plagiarism; manipulation of results or images; authorship fraud; fake peer review (i.e., submission of fabricated reviewer reports, often via falsified reviewer identities); salami slicing (i.e., division of one substantial study into multiple smaller publications to inflate output); use of paper mills (i.e., manuscripts produced by third-party organizations that sell fraudulent research); ethical violations; or sabotage of materials. The full list of criteria is available in the Supplementary Material (S1 Appendix). This classification method has been applied in prior studies investigating retraction causes [7,10]. Records with missing retraction reasons were excluded from misconduct-specific analyses but were retained for general gender analyses.

### Gender assignment

Gender was inferred using Gender API (https://gender-api.com), a service that predicts binary gender (male/female) based on first names and provides a confidence score [37]. For each author, the first name was extracted. Authors with a single-letter first name were excluded, as were those for whom Gender API provided no prediction or a confidence score below the inclusion thresholds. Unisex or ambiguous names were automatically assigned lower confidence scores by Gender API and therefore frequently fell below our inclusion thresholds.

Two datasets were created based on gender assignment confidence: one including authors with confidence ≥60%, and another limited to confidence ≥70%. The ≥ 60% sample served as the basis for the main analyses, while the ≥ 70% sample was used for sensitivity checks. Analyses were conducted separately for both datasets, following the methodology of prior studies using algorithmic gender inference [7,18,20]. Gender was determined for all authors listed in each retracted article, as well as separately for first and last authors, using the full unprocessed names as they appeared in the dataset. To assess inference accuracy, we manually verified the gender classification for a random sample of 200 names and found no misclassifications.

To determine the number of unique authors, we standardized names to improve matching. Standardization involved (i) trimming leading/trailing spaces, (ii) removing punctuation, hyphens, and parentheses, (iii) converting to lowercase, and (iv) removing diacritical marks. Authors were then classified based on last and first names. We manually reviewed potential duplicates in which the same first and last name appeared with variations in intermediate names or initials, and considered them the same individual if they were affiliated with the same institution.

In total, 807 retracted articles included at least one author name. These articles contained 4,136 individual author entries, of which 3,909 included full first names (i.e., no initials). Gender could be assigned to 3,865 of these authors (98.9%). A total of 3,743 authors met the 60% confidence threshold, and 3,555 met the 70% threshold. The 4,136 authorships corresponded to 2,864 unique individuals, among whom 2,663 had full first names. Gender was assigned to 2,621 unique authors (98.4%), with 2,505 and 2,329 meeting the 60% and 70% confidence thresholds, respectively. The full anonymized dataset for the 2,663 unique authors is available as Supporting Information (S1 Data).

For first authorship analysis, we identified 772 retracted publications with first authors having full first names. Gender could be inferred for 767 of them, with 747 meeting the 60% and 721 the 70% threshold. For last authors, 701 had full first names, and gender could be assigned to 697 (687 at 60% and 669 at 70%). The high match rates observed for Gender API are consistent with previous research reporting that the proportion of non-classifications (‘naCoded’) can be as low as 0.34% [37].

### Statistical analyses

We computed the proportion of male and female authors among all retracted publications, and separately for first and last authors. We repeated these calculations for the subset of retractions related to misconduct, allowing comparisons between overall retractions and misconduct-specific retractions. We also examined gender differences in the number of retracted publications per author.

To test differences in retraction volume by gender, we used the Wilcoxon rank-sum test to compare the median number of retracted publications per author between men and women. Authors were also grouped by number of retractions (1, 2–5, and >5), and gender distributions across these categories were compared using the chi-squared test.

We then stratified the dataset by clinical discipline and calculated the proportion of male and female first and last authors per specialty. These proportions were compared with data from a previously published study by Hart & Perlis, which analyzed gender representation in biomedical authorship across ten medical specialties for the years 2008–2017 [18]. Table 2 summarizes key parameters of our dataset alongside the Hart & Perlis data, providing context for comparing female authorship in retracted publications with its overall representation. For comparability across disciplines, we restricted our retraction dataset to 2008–2017, consistent with Hart & Perlis.

**Table 2:** Comparison of methods used to collect retraction data and publication benchmark data (publication data from Hart & Perlis [18]).

Parameter	Retraction data	Publication benchmark data	Notes
Data sources	Retraction Watch Database (RWD), PubMed, Web of Science (WoS)	PubMed	Using only PubMed would have identified 758 (instead of 878) retracted publications
Journal selection	Top 15 journals by Journal Citation Reports (JCR) impact factor in each of nine medical disciplines	Same as retraction data, plus a cross-disciplinary field (general medical journals)	The cross-disciplinary field was excluded from our analysis to ensure comparability
Time frame	From inception to 2024	2008-2017	For comparison, retraction data were restricted to 2008–2017
Number of articles	878 retracted publications	274,764 publications	
Gender assignment tool	Gender API	Genderize.io	
Gender prediction thresholds	≥60 (primary dataset) and ≥70% (secondary dataset)	≥60%	
Proportion of authors with identified gender	91% (3,743/ 4,136) at ≥60%; 86% (3,555/ 4,136) at ≥70%	78% (1,536,026/ 1,981,454)	

Statistical analyses were performed using Stata version 15.1. The study followed STROBE (Strengthening the Reporting of Observational Studies in Epidemiology) guidelines.

### Ethics statement

This bibliometric study relied exclusively on published records and did not involve patient or personal data. Therefore, ethical approval was not required under Swiss law. Author names were part of the publication metadata used for analysis, but these identifying data have been removed from the Supporting Information file prior to publication to ensure compliance with PLOS ONE’s data sharing policy.

## Results

A total of 878 retracted publications were identified among 422,827 publications across 131 high-impact journals spanning nine medical disciplines, corresponding to a retraction rate of 2.08 per 1,000 publications. Among the 811 retracted publications with available data on reasons, 66.8% were attributed to misconduct.

**Fig 1:**
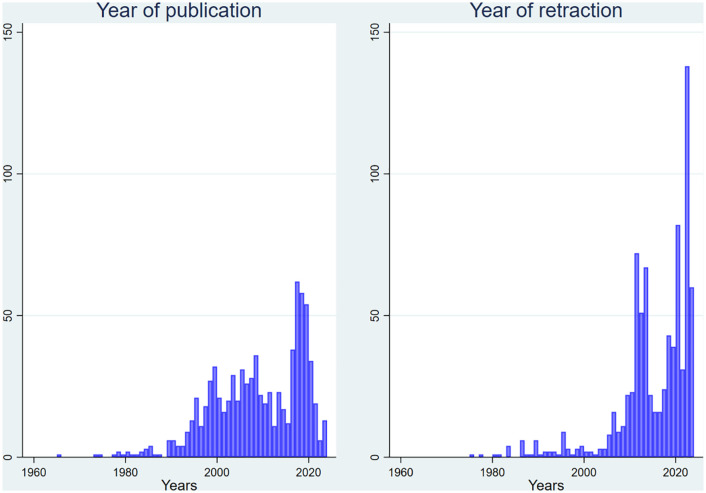
Publication and retraction years of 878 retracted publications from 131 high-impact journals across nine medical disciplines.

**Fig 2:**
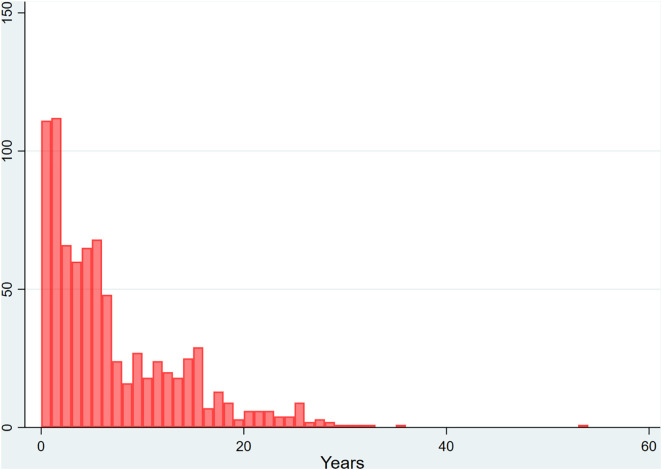
Delay in years between publication and retraction for 878 retracted publications from 131 high-impact journals across nine medical disciplines.

Fig 1 shows the distribution of publication and retraction years for these 878 retracted publications. Articles were published between 1965 and 2024, with a median publication year of 2009 (interquartile range [IQR]: 16 years), and retracted between 1975 and 2024, with a median retraction year of 2017 (IQR: 10 years). Fig 2 illustrates the delay between publication and retraction, which ranged from 0 to 54 years (median: 4 years, IQR: 9). Other results not directly related to gender—including author count per article, article types, countries of affiliation, and retraction patterns by journal and discipline—are presented in a separate article submitted from the same project.

The gender distribution varied depending on whether all author entries or unique individuals were considered. Among all authors with gender prediction confidence ≥60%, 2,880 (76.9%) were identified as men and 863 (23.1%) as women. When restricted to unique individuals, men represented 69.1% (n = 1,732) and women 30.9% (n = 773).

Disparities were more pronounced when focusing on first and last authors. Among first authors, 83.5% of all entries were men and 16.5% women; for unique individuals, the breakdown was 70.5% and 29.5%. For last authors, men accounted for 87.3% of all entries and 79.1% of unique individuals.

Gender gaps widened when considering only publications retracted for misconduct. Among first authors, men made up 88.5% of all entries and women 11.5%. For last authors, men represented 90.4% and women 9.6%.

**Fig 3:**
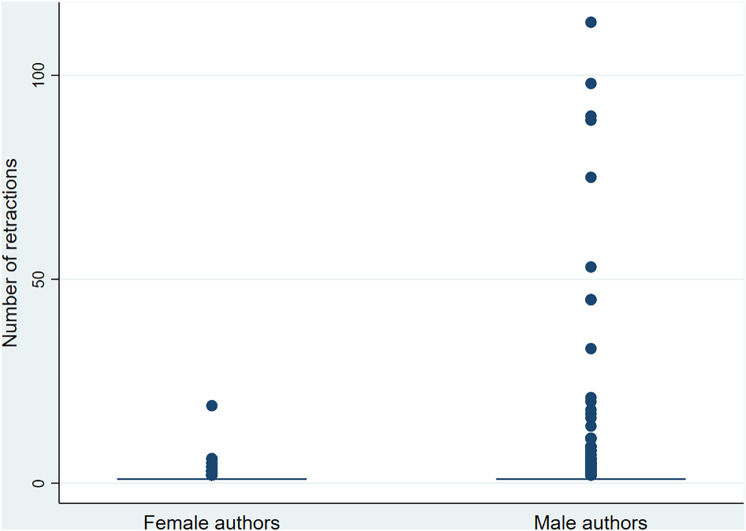
Box plot of the number of retractions per author, by gender, at a gender prediction confidence threshold of 60%, based on 878 retracted publications from 131 high-impact medical journals (n = 2,505 unique authors: 1,732 men and 773 women).

**Fig 4:**
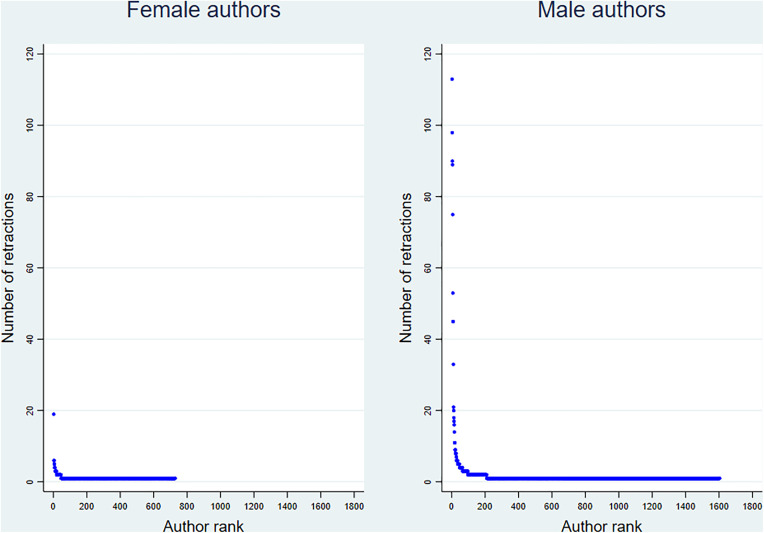
Scatter plot showing the number of retractions per author by gender, at a gender prediction confidence threshold of 60%, based on 878 retracted publications from 131 high-impact medical journals (n = 2,505 unique authors: 1,732 men and 773 women). Authors are sorted on the x-axis by number of retractions, from highest to lowest.

Gender differences were also apparent across categories of retraction frequency (Table 3). Men were significantly more likely than women to have multiple retractions across all authorship positions. For instance, among those with more than five retracted publications (n = 37), 91.9% were men and only 8.1% were women. Although the median number of retractions was identical by gender (1; IQR = 0), counts differed significantly between men and women. Figs 3 and 4 further illustrate these patterns. Fig 3 presents a box plot showing the number of retractions per author by gender, while Fig 4 displays a scatter plot ranking authors by number of retractions and gender.

**Table 3:** Gender distribution by author position and retraction count, at a gender prediction confidence threshold of 60%, based on 878 retracted publications from 131 high-impact medical journals (n = 2,864 unique authors, of whom 2,663 had full first names and 2,621 could be assigned a gender).

Gender	Number of authors with ≥1 retracted publication (%)	Number of authors with 1 retracted publication (%)	Number of authors with 2–5 retracted publications (%)	Number of authors with >5 retracted publications (%)	p-value^1^	Median number of retracted publications (IQR)	Min-max	p-value^2^
All authors	2505 (100)	2245 (100)	223 (100)	37 (100)	<0.001	1 (0)	1-113	<0.001
Women	773 (30.9)	729 (32.5)	41 (18.4)	3 (8.1)		1 (0)	1-19	
Men	1732 (69.1)	1516 (67.5)	182 (81.6)	34 (91.9)		1 (0)	1-113	
All first authors	376 (100)	329 (100)	36 (100)	11 (100)	0.03	1 (0)	1-107	0.02
Women	111 (29.5)	104 (31.6)	7 (19.4)	0		1 (0)	1-5	
Men	265 (70.5)	225 (68.4)	29 (80.6)	11 (100)		1 (0)	1-107	
All last authors	387 (100)	332 (100)	44 (100)	11 (100)	0.01	1 (0)	1-98	0.002
Women	81 (20.9)	78 (23.5)	3 (6.8)	0		1 (0)	1-5	
Men	306 (79.1)	254 (76.5)	41 (93.2)	11 (100)		1 (0)	1-98	

^1^Chi-squared test comparing the distribution of male and female authors across retraction count categories (1, 2–5, > 5).

^2^Wilcoxon rank-sum test comparing the median number of retracted publications between male and female authors.

Tables 4 and 5 present the proportion of women among first and last authors by discipline for both the full retraction sample and the subset of retractions due to misconduct. For comparability with publication benchmarks reported by Hart & Perlis [18]—who estimated that, in 2008–2017, women accounted overall for 41.3–45.4% of first authors and 26.1–33.4% of last authors, and across specialties for 31.0–59.2% of first authors and 17.7–44.4% of last authors—we also report retraction data restricted to 2008–2017.

**Table 4:** Proportion of women among first authors by discipline, for all retracted publications and those retracted for misconduct, based on 878 retracted publications from 131 high-impact journals across nine medical disciplines (disciplines listed in alphabetical order). Data are shown for names with gender prediction confidence ≥60%.

Discipline	Total retracted publications with identified gender, n	Women as first authors of retracted publications, n (%)	Women as first authors of retracted publications for 2008–2017, n (%)	Women as first authors of publications for 2008–2017, %^1^	Retracted publications for misconduct with identified gender, n	Women as first authors of misconduct-related retractions, n (%)
ANESTHESIOLOGY (n = 382)	366	25 (6.8)	10 (10.2)	33.5-36.7	331	19 (5.7)
CLINICAL NEUROLOGY (n = 62)	43	6 (14.0)	2 (16.7)	38.3-41.4	17	1 (5.9)
DERMATOLOGY (n = 18)	13	8 (61.5)	4 (80.0)	48.9-51.8	3	1 (33.3)
MEDICINE, GENERAL & INTERNAL (n = 125)	105	21 (20.0)	7 (24.1)	34.2-42.1	43	7 (16.3)
OBSTETRICS & GYNECOLOGY (n = 116)	69	16 (23.2)	4 (11.1)	50.0-59.2	36	7 (19.4)
ONCOLOGY (n = 92)	73	23 (31.5)	13 (41.9)	45.0-46.6	42	14 (33.3)
PEDIATRICS (n = 20)	15	4 (26.7)	0	54.5-58.6	7	1 (14.3)
PSYCHIATRY (n = 44)	39	15 (38.5)	7 (35.0)	42.3-44.7	23	6 (26.1)
RADIOLOGY, NUCLEAR MEDICINE & MEDICAL IMAGING (n = 33)	24	5 (20.8)	4 (40.0)	31.0-36.8	12	3 (25.0)
Total (n = 892)^2^	747^3^	123 (16.5)	51 (20.7)	41.3-45.4	514	59 (11.5)

^1^Publication benchmarks were based on Hart & Perlis, who examined the proportion of women as first and last authors in 134 journals across 10 medical specialties for the years 2008–2017 [18].

^2^The total number of retracted publications sums to 892 (not 878) because four journals were assigned to two disciplines: *J Am Acad Child Adolesc Psychiatry* (PEDIATRICS and PSYCHIATRY), *J Neurol Neurosurg Psychiatry* (CLINICAL NEUROLOGY and PSYCHIATRY), *Neuro Oncol* (CLINICAL NEUROLOGY and ONCOLOGY), and *Ultrasound Obstet Gynecol* (OBSTETRICS & GYNECOLOGY and RADIOLOGY, NUCLEAR MEDICINE & MEDICAL IMAGING).

^3^The number of retracted publications sums to 747 (not 892) due to missing data: articles with no identified first author or undetermined gender (i.e., abbreviated first names or gender prediction confidence <60%).

**Table 5:** Proportion of women among last authors by discipline, for all retracted publications and those retracted for misconduct, based on 878 retracted publications from 131 high-impact journals across nine medical disciplines (disciplines listed in alphabetical order). Data are shown for names with gender prediction confidence ≥60%.

Discipline	Total retracted publications with identified gender, n	Women as last authors of retracted publications, n (%)	Women as last authors of retracted publications for 2008–2017, n (%)	Women as last authors of publications for 2008–2017, %^1^	Retracted publications for misconduct with identified gender, n	Women as last authors of misconduct-related retractions, n (%)
ANESTHESIOLOGY (n = 382)	332	14 (4.2)	4 (4.2)	23.7-26.0	300	11 (3.7)
CLINICAL NEUROLOGY (n = 62)	44	6 (13.6)	2 (14.3)	23.6-28.8	17	2 (11.8)
DERMATOLOGY (n = 18)	9	2 (22.2)	0	29.2-37.4	2	0
MEDICINE, GENERAL & INTERNAL (n = 125)	98	22 (22.5)	4 (14.3)	23.3-32.1	40	8 (20.0)
OBSTETRICS & GYNECOLOGY (n = 116)	63	17 (27.0)	7 (24.1)	31.0-44.4	38	10 (26.3)
ONCOLOGY (n = 92)	70	14 (20.0)	8 (25.8)	24.9-32.7	43	9 (20.9)
PEDIATRICS (n = 20)	15	7 (46.7)	3 (50.0)	37.0-42.6	7	3 (42.9)
PSYCHIATRY (n = 44)	36	3 (8.3)	2 (11.8)	28.3-34.0	23	2 (8.7)
RADIOLOGY, NUCLEAR MEDICINE & MEDICAL IMAGING (n = 33)	20	2 (10.0)	1 (12.5)	17.7-25.3	11	1 (9.1)
Total (n = 892)^2^	687^3^	87 (12.7)	31 (13.4)	26.1-33.4	481	46 (9.6)

^1^Publication benchmarks were based on Hart & Perlis, who examined the proportion of women as first and last authors in 134 journals across 10 medical specialties for the years 2008–2017 [18].

^2^The total number of retracted publications sums to 892 (not 878) because four journals were assigned to two disciplines: *J Am Acad Child Adolesc Psychiatry* (PEDIATRICS and PSYCHIATRY), *J Neurol Neurosurg Psychiatry* (CLINICAL NEUROLOGY and PSYCHIATRY), *Neuro Oncol* (CLINICAL NEUROLOGY and ONCOLOGY), and *Ultrasound Obstet Gynecol* (OBSTETRICS & GYNECOLOGY and RADIOLOGY, NUCLEAR MEDICINE & MEDICAL IMAGING).

^3^The number of retracted publications sums to 687 (not 892) due to missing data: articles with no identified first author or undetermined gender (i.e., abbreviated first names or gender prediction confidence <60%).

Overall, and in nearly all specialties, the proportion of women in these authorship positions was substantially lower than the corresponding publication benchmarks, with the gender disparity even more pronounced in retractions related to misconduct. For instance, among 366 retracted publications with identifiable first authors in anesthesiology, just 6.8% had female first authors (10.2% when limited to 2008–2017). Likewise, among 332 anesthesiology papers with identified last authors, only 4.2% were female, both overall and for 2008–2017. When restricting the analysis to retractions due to misconduct, the female underrepresentation was even stronger, with women accounting for only 5.7% of first authors and 3.7% of last authors. By contrast, Hart & Perlis reported that women accounted for 33.5–36.7% of first authors and 23.7–26.0% of last authors in anesthesiology publications.

Dermatology and pediatrics were the only two disciplines in which the proportion of women among retracted authors exceeded the publication benchmarks in both the whole retraction sample and the subset restricted to 2008–2017. In dermatology, women accounted for 61.5% of first authors in the whole sample and 80.0% in 2008–2017, both above the estimated 48.9–51.8% benchmark for female first authors. In pediatrics, women represented 46.7% of last authors in the whole sample and 50.0% in 2008–2017, compared with an expected range of 37.0–42.6%. A similar, though less consistent, pattern was observed in radiology, where women represented 40.0% of first authors of retracted articles in 2008-2017, exceeding the 31.0-36.8% benchmark, although their proportion over the entire study period was lower (20.8%). The findings for these disciplines are based on small numbers of retracted articles and should therefore be interpreted with caution.

### Secondary analyses (gender assignment confidence ≥70%)

Results at the ≥ 70% confidence threshold closely mirrored those obtained at ≥60%. Overall, 2,738 authors (77.0%) were identified as men and 817 (23.0%) as women; among unique individuals, men accounted for 68.8% (n = 1,602) and women 31.2% (n = 727).

Gender gaps were greater among senior authorship positions: 83.9% of first authors and 87.7% of last authors were men, compared with 16.1% and 12.3% women, respectively; among unique individuals, men represented 70.4% of first authors and 79.4% of last authors. Disparities were even wider in misconduct-related retractions, with men comprising 88.6% of first authors and 90.9% of last authors. Men were also more likely to accumulate multiple retractions (S1 Table).

## Discussion

### Summary of the findings

In this cross-sectional study of 878 articles retracted from 131 high-impact medical journals, we examined gender disparities among their authors. Women were consistently underrepresented, particularly in first and last authorship positions, and this disparity was even more pronounced in retractions related to misconduct. Women were also significantly less likely to have multiple retractions. These gender disparities were observed across most disciplines, with the exception of dermatology and radiology among first authors, and pediatrics among last authors, where the proportion of female authors of retracted publications exceeded general authorship benchmarks—though these findings were based on small sample sizes.

### Comparison with existing literature

Our findings are consistent with prior literature examining gender and retractions. In a preliminary study by our research team, we examined 438 retracted publications in medicine and found that women represented only 25% of first and 14% of last authors, with even lower proportions in misconduct-related cases [7]. Pinho-Gomes et al. conducted a broader analysis of over 35,000 retracted biomedical publications and similarly reported that women were significantly underrepresented—accounting for 27% of first and 24% of last authors overall, and only 19% and 14%, respectively, in fraud-related retractions [14]. Decullier & Maisonneuve analyzed a smaller sample of 113 retractions and found that misconduct (fraud or plagiarism) was significantly more frequent among male-authored publications (59%) than among those authored by women (29%) [13]. Notably, neither of the latter two studies included a comparison group reflecting the general gender distribution among all publications. More recently, Zheng et al. confirmed male overrepresentation in retractions across multiple disciplines, with patterns varying by field [15], while Maddi et al. highlighted the role of team composition, showing higher retraction risk in mixed-gender teams [16].

Together, these studies—including our own—suggest that women are consistently underrepresented among authors of retracted publications, particularly those retracted for misconduct. These gendered patterns may reflect broader inequalities in academic positions, authorship roles, and exposure to investigative or editorial scrutiny. However, further research is needed to disentangle potential drivers such as behavioral, cultural, or systemic factors, including gender bias in retraction practices themselves.

Several studies have examined the reasons for retraction and patterns of retraction across disciplines and countries. Fang et al. found that misconduct accounts for the majority of retractions (67%), a finding consistent with our study, where the same proportion of retractions were attributed to misconduct [2,38]. A recent analysis by our research group comparing the performance of three major bibliographic databases (RWD, PubMed, WoS Core collection) showed inconsistencies in retraction indexing and emphasized the importance of using multiple sources to obtain comprehensive retraction data [1]. We used this same dataset, enhanced with gender inference, to explore author-level characteristics.

### Implications for practice and research

The underrepresentation of women among retracted authors, particularly for misconduct-related retractions, may reflect systemic gender imbalances in academia rather than differences in scientific integrity. Women continue to be underrepresented in senior academic positions [21,23], which may reduce both their visibility and their vulnerability to scrutiny or allegations of misconduct. Alternatively, it is also possible that the types of research or positions held by women expose them to fewer opportunities for retraction-inducing misconduct.

Our findings underscore the importance of context when interpreting retraction data. Retractions are not only about correcting the literature but also about understanding broader issues of research culture, responsibility, and inequality. Bibliometric analyses should consider demographic variables, including gender, to ensure that corrective mechanisms do not disproportionately affect certain groups.

Future research should explore how institutional policies, peer review practices, and editorial oversight may contribute to observed disparities. Qualitative studies could also help understand the social and professional dynamics that lead to retractions, including gendered experiences of scrutiny, pressure, or misconduct allegations.

### Limitations

Our study has several limitations. First, gender was inferred algorithmically, which may not accurately reflect individuals’ self-identified gender. Although we used two confidence thresholds (≥60% and ≥70%), some misclassification is possible. Second, we excluded authors with abbreviated or ambiguous first names, which may introduce selection bias. Third, retraction reasons were classified based on metadata, and the accuracy of these classifications can vary across journals and time periods. Furthermore, our analysis focused only on high-impact journals, which may not reflect gender disparities in lower-impact or non-English-language journals. Fourth, while our comparisons with Hart & Perlis inherently account for discipline and publication year, we could not adjust for other potential confounders such as team size, open access status, or geographic region, which may influence publishing patterns and retraction dynamics. Fifth, although we applied standardization and manual checks to identify unique authors, minor errors in disambiguation cannot be excluded; however, this metric was a secondary outcome and is unlikely to affect our main findings. Finally, while our findings show associations between gender and retraction patterns, they do not establish causality.

## Conclusion

This study demonstrates that women are underrepresented among authors of retracted publications in high-impact medical journals, particularly in misconduct-related cases and in key authorship positions. These disparities were observed consistently across most medical disciplines and align with broader patterns of gender imbalance in academic publishing.

Although this study did not aim to explore the reasons behind these differences, the findings underscore the importance of further research to understand the underlying factors. A better understanding of the social, institutional, and editorial dynamics surrounding retractions could help ensure that the scientific correction process is both rigorous and equitable.

## Supporting information

S1 Appendix Criteria for identifying misconduct-related retractions using Retraction Watch Database. (DOCX)

S1 Data Anonymized dataset of 2,663 unique authors of retracted publications from 131 high-impact medical journals (n = 878 articles). Includes gender assignment (ga_gender), confidence score from Gender API (ga_accuracy), and number of samples used for the gender inference (ga_samples). All personally identifiable information has been removed.
(XLSX)

S1 Table Gender distribution by author position and retraction count, at a gender prediction confidence threshold of 70%, based on 878 retracted publications from 131 high-impact medical journals (n = 2,864 unique authors, of whom 2,663 had full first names and 2,621 could be assigned a gender). (DOCX)
