# Supplementary material for: Gender disparities among authors of retracted publications in medical journals: A cross-sectional study
Source: PLoS One. 2025 Nov 19;20(11):e0335059. doi: 10.1371/journal.pone.0335059 (PMC12629481; doi:10.1371/journal.pone.0335059)
Supplement: S1 Appendix — (DOCX) [file pone.0335059.s001.docx]

S1_Appendix. Criteria for identifying misconduct-related retractions using Retraction Watch Database

+Author Unresponsive OR +Breach of Policy by Author OR +Concerns/Issues About Authorship OR +Conflict of Interest OR +Duplication of Article OR +Duplication of Data OR +Duplication of Image OR +Duplication of Text OR +Ethical Violations by Author OR +Fake Peer Review OR +Falsification/Fabrication of Data OR +Falsification/Fabrication of Image OR +Falsification/Fabrication of Results OR +False/Forged Authorship OR +Informed/Patient Consent - None/Withdrawn OR +Lack of Approval from Author OR +Lack of IRB/IACUC Approval OR +Manipulation of Images OR +Manipulation of Results OR +Miscommunication by Author OR +Misconduct by Author OR +Nonpayment of Fees/Refusal to Pay OR +Original Data not Provided OR +Paper Mill OR +Plagiarism of Article OR +Plagiarism of Data OR +Plagiarism of Image OR +Plagiarism of Text OR +Randomly Generated Content OR +Sabotage of Materials OR +Salami Slicing
