# Supplementary material for: Gender disparities among authors of retracted publications in medical journals: A cross-sectional study
Source: PLoS One. 2025 Nov 19;20(11):e0335059. doi: 10.1371/journal.pone.0335059 (PMC12629481; doi:10.1371/journal.pone.0335059)
Supplement: S1 Table — (DOCX) [file pone.0335059.s003.docx]

S1_Table. Gender distribution by author position and retraction count, at a gender prediction confidence threshold of 70%, based on 878 retracted publications from 131 high-impact medical journals (n=2,864 unique authors, of whom 2,663 had full first names and 2,621 could be assigned a gender).

| Gender | Number of authors with ≥1 retracted publication (%) | Number of authors with 1 retracted publication (%) | Number of authors with 2-5 retracted publications (%) | Number of authors with >5 retracted publications (%) | p-value^1^ | Median number of retracted publications (IQR) | Min-max | p-value^2^ |
| --- | --- | --- | --- | --- | --- | --- | --- | --- |
| All authors | 2329 (100) | 2077 (100) | 215 (100) | 37 (100) | <0.001 | 1 (0) | 1-113 | <0.001 |
| Women | 727 (31.2) | 683 (32.9) | 41 (19.1) | 3 (8.1) |  | 1 (0) | 1-19 |  |
| Men | 1602 (68.8) | 1394 (67.1) | 174 (80.9) | 34 (91.9) |  | 1 (0) | 1-113 |  |
| All first authors | 351 (100) | 305 (100) | 35 (100) | 11 (100) | 0.03 | 1 (0) | 1-107 | 0.02 |
| Women | 104 (29,6) | 97 (31.8) | 7 (20.0) | 0 |  | 1 (0) | 1-5 |  |
| Men | 247 (70.4) | 208 (68.2) | 28 (80.0) | 11 (100) |  | 1 (0) | 1-107 |  |
| All last authors | 369 (100) | 314 (100) | 44 (100) | 11 (100) |  | 1 (0) | 1-98 | 0.003 |
| Women | 76 (20.6) | 73 (23.3) | 3 (6.8) | 0 |  | 1 (0) | 1-5 |  |
| Men | 293 (79.4) | 241 (76.8) | 41 (93.2) | 11 (100) |  | 1 (0) | 1-98 |  |

^1^ Chi-squared test comparing the distribution of male and female authors across retraction count categories (1, 2–5, >5).

^2^ Wilcoxon rank-sum test comparing the median number of retracted publications between male and female authors.
